# Effect of specific surface area on syngas production performance of pure ceria in high-temperature thermochemical redox cycling coupled to methane partial oxidation

**DOI:** 10.1039/d0ra06280d

**Published:** 2020-10-06

**Authors:** Manabu Heya, Xiang Gao, Antonio Tricoli, Wojciech Lipiński

**Affiliations:** Department of Electronics, Information and Communication Engineering, Faculty of Engineering, Osaka Sangyo University 3-1-1 Nakagaito Daito-city Osaka 574-8530 Japan heya@eic.osaka-sandai.ac.jp +81 72 875 3001; ASU LightWorks®, Arizona State University Tempe AZ 85287-5402 USA xiang.gao.10@asu.edu +1 480 276 2714; Nanotechnology Research Laboratory, Research School of Electrical, Energy and Materials Engineering, The Australian National University Canberra ACT 2601 Australia antonio.tricoli@anu.edu.au +61 2 612 51696; Solar Thermal Group, Research School of Electrical, Energy and Materials Engineering, The Australian National University Canberra ACT 2601 Australia wojciech.lipinski@anu.edu.au +61 2 612 57896

## Abstract

Specific surface area is a key parameter determining the rates of thermochemical redox reactions in metal oxides. We have experimentally investigated the effect of specific surface area on syngas production of pure ceria powders under two experiments such as a heating experiment without syngas production and an isothermal thermochemical redox cycling experiment using carbon dioxide splitting and methane partial oxidation. The specific surface area of ceria powders decreased relatively slowly during 50 hours of ceria powder heating without syngas production due to a combination of oriented attachment and grain-boundary diffusion. When cycled thermochemically, the specific surface area of ceria powders rapidly decreased only in the initial 10 minutes of reduction in the 1st cycle due to evaporation and condensation. A significant decrease of specific surface area during the initial stage of thermochemical ceria powder cycling is unavoidable even if temperatures as low as *T* = 1173 K are used in the reduction reaction coupled to methane partial oxidation.

## Abstract 2

Specific surface area is a key parameter determining the rates of thermochemical redox reactions in metal oxides.

## Graphical abstract

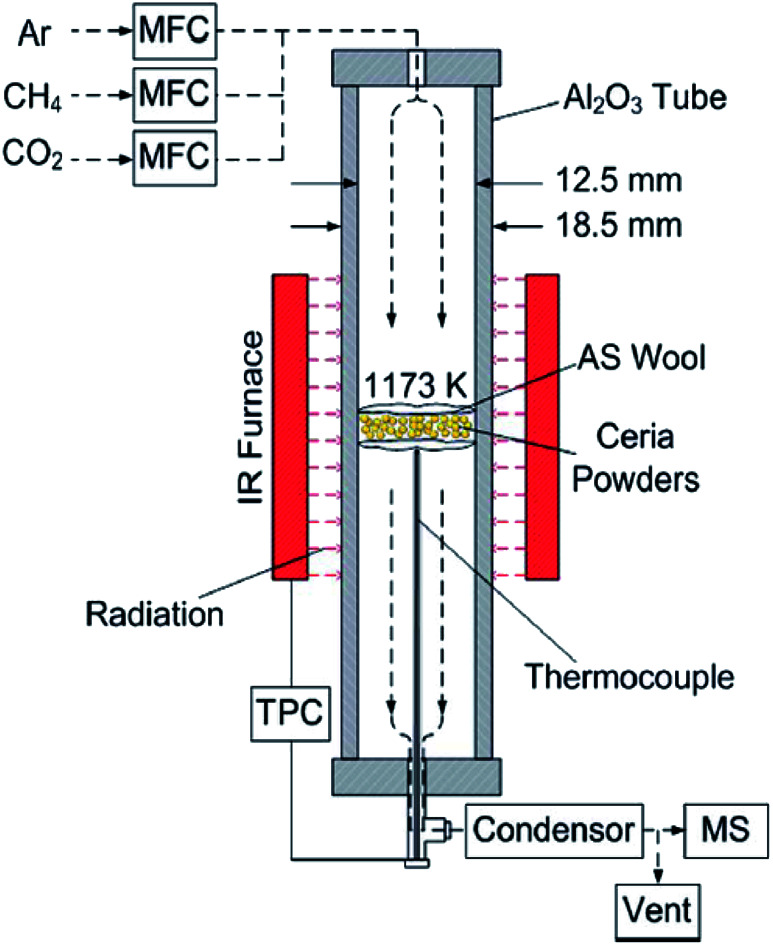

## Introduction

Synthesis gas (syngas) production *via* high-temperature metal oxide based thermochemical redox cycling driven by concentrated solar radiation is a promising method for chemical storage of solar energy.^1–3^ A two-step redox cycling can be written as,

Solar, endothermic reduction step:

1 (1/*δ*)MO_2_ → (1/*δ*)MO_2−*δ*_ + (1/2)O_2_ (g)

Non-solar, exothermic oxidation step:

2a (1/*δ*)MO_2−*δ*_ + H_2_O (g) → (1/*δ*)MO_2_ + H_2_ (g)

2b (1/*δ*)MO_2−*δ*_ + CO_2_ (g) → (1/*δ*)MO_2_ + CO (g)

Net H_2_O dissociation:

3a H_2_O (g) → (1/2)O_2_ (g) + H_2_ (g)

Net CO_2_ dissociation:

3b CO_2_ (g) → (1/2)O_2_ (g) + CO (g)

where M is a metal (Ce in the present study) and *δ* is the oxygen non-stoichiometry in a metal oxide.

The cycle (1)–(2) has been demonstrated with different oxygen exchange materials under temperature-swing and isothermal conditions.^3–7^ Typical temperatures for the reduction and oxidation steps are above 1700 K and below 1400 K, respectively. The temperature swing between the reduction and oxidation steps necessitates application of heat recovery strategies to maximize process efficiency, and leads to considerable thermal stresses.^8^ In an ideal isothermal cycling, the temperature swing between the two steps is eliminated.^9–11^ Alternatively, the reduction reaction (1) can be carried out under carbothermal conditions, which allows for lowering the reduction temperature and attaining higher non-stoichiometry in the metal oxide.^3,12^ For methane as the reducing carbonaceous material, the non-stoichiometric reduction reaction of ceria coupled to methane partial oxidation (MPO) can be written as:^3,12^

4 (1/*δ*)CeO_2_ + CH_4_ (g) → (1/*δ*)CeO_2−*δ*_ + CO (g) + 2H_2_ (g)

High specific surface area (SSA) in porous structures of oxygen exchange materials is a key requirement to enable high oxidation rates,^1,13^ low pressure drop^11,14^ and enhanced heat and mass transfer,^11,14,15^ which in turn result in increased efficiency of syngas production. The effect of the morphology of pure ceria samples, especially their SSA, to enhance syngas production were investigated for three-dimensionally ordered macro-porous (3DOM) ceria structures,^13,16,17^ reticulated porous ceramics,^15^ wood-templated structures,^18^ fibrous structures^19,20^ and nanostructured powders.^21^ In these studies except ref. 18 and 21, temperatures *T* > 1500 K were used in the reduction step, for which a rapid sintering and a remarkable decrease of SSA of ceria samples were reported.^20^

Ceria-based isothermal redox cycling with the reduction step under an inert gas atmosphere was studied in ref. 10 and 16. As the temperature of the entire cycle is set by the minimum required reduction temperature, typically at *T* > 1500 K, this approach results in a significant sintering of ceria, difficulties in manufacturing industry-scale reactors and low solar-to-fuel efficiency even with considerable gas-phase heat recovery.^10,14^ In order to lower the cycle temperature below 1273 K, the metal-oxide reduction reaction can be coupled to MPO as given by eqn (4).^3–7,12^ Gao *et al.* synthesized three types of ceria morphologies with different SSA and porosities. The materials were comparatively investigated for their initial and long-term syngas production performances during the isothermal redox cycling coupled to MPO at the reduction step and carbon dioxide splitting (CDS) at the oxidation step.^21^ Nanostructured flame-made agglomerates with the highest SSA showed an enhanced syngas production. Specifically, the time-averaged production rate at the 10th cycle for nanostructured flame-made agglomerates was at most ∼57% higher than that of commercial micron-sized ceria powders, although the SSA of nanostructured flame-made agglomerates after the 10th cycle was 4 times higher than that of commercial ceria powders. Thus, the effect of SSA on syngas production rate (SPR) and the sintering mechanism in the isothermal redox cycling of ceria coupled to MPO and CDS have not been fully understood yet.

Sintering mechanism of pure and doped ceria under high temperatures without syngas production has been reported in the literatures.^22–33^ In this study, we experimentally investigate the effect of specific surface area on syngas production of pure ceria powders during heating without syngas production (further referred to as heating experiments) and during an isothermal MPO–CDS thermochemical redox cycling (further referred to as cycling experiments). The temperatures are varied in the range 973–1773 K in the heating experiments, whereas the reduction and oxidation temperatures are fixed at 1173 K in the cycling experiments. Additionally, morphological and crystal changes based on the results observed by a scanning electron microscopy (SEM images) and an X-ray diffraction (XRD spectra) are also shown as a reference.

## Experimental

**Fig. 1:**
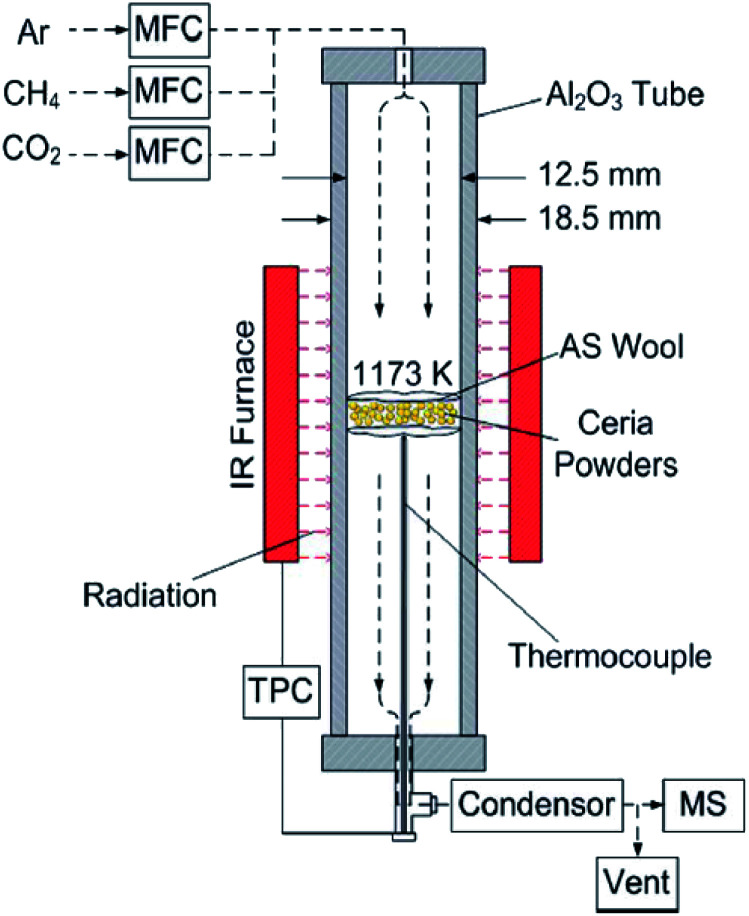
Schematics of the experimental set-up using an IR furnace for cycling experiments.^21^ MFC: mass flow controller; TPC: temperature program controller; MS: mass spectrometer; AS wool: aluminosilicate wool.

Heating experiments are conducted using an electric furnace (Laboratory Furnaces, LABEC Ltd). Cycling experiments are conducted using the setup shown in Fig. 1, which includes a vertical-tube reactor (alumina tube) placed inside an electric IR furnace (P4C-VHT, Advance Riko Ltd.).^4–7,21^ Commercial pure ceria powders with a typical diameter of 5 μm (REacton®, 99.9% (REO), 11 328, LOT: T16A086, Alfa Aesar Ltd.) are used as the sample material in both heating experiments and cycling experiments. The initial SSA for the as-received samples is obtained by calculating an average SSA for nine as-received samples, and is 8.02 ± 0.80 cm^2^ g^−1^. Particle size distribution is as follows: *d*_10_ = 3.04 μm, *d*_50_ = 5.61 μm, *d*_50_ = 10.34 μm, and *d*_mean_ = 6.30 μm, where *d*_mean_ is a mean diameter.

### Heating experiment

Three ceria powder samples of ∼300 mg are placed in alumina crucibles and heated using the electric furnace under atmospheric air conditions. Dwell temperatures and dwell times are varied in the ranges of 973–1773 K and 1/60–75 h in these experiments, respectively. The temperature is programmed as: (1) linear temperature increase from room temperature to dwell temperatures at a constant rate of 5 K min^−1^ and (2) constant dwell temperature during the dwell time. Note that experimental results from SSA and X-ray measurements after heating experiments are obtained by averaging three values for a given experimental condition.

## Cycling experiment

The experimental set-up used in cycling experiments is shown in Fig. 1, and is the same as that used in ref. 21. The ceria powder samples of ∼300 mg are packed between two pieces of 2 mm thick highly porous and temperature-resistant aluminosilicate wool inside the vertical-tube reactor of Fig. 1. The ceria powders are heated in the vertical-tube reactor placed inside the IR furnace. Gas mixtures are regulated by mass flow controllers (MFC, F-201CV, Bronkhorst) before being delivered through the top of the tube. Sample temperature is measured using an alumina sealed type-K thermocouple inside the alumina tube. The composition of the product gases is continuously monitored by using a quadrupole mass spectrometer (OmniStar™ GSD320, Pfeiffer Vacuum). All the monitored gas components are calibrated in the mass spectrometer using standard mixtures of the individual solute (CH_4_, CO_2_ and CO/H_2_) in an Ar carrier gas.

The reduction and oxidation temperatures are fixed at 1173 K. This temperature was selected based on the previous studies in which the temperature range 973–1273 K was used with the experimental setup shown in Fig. 1 in order to produce H_2_ and CO with the molar ratio of 2 : 1 while maintaining sufficient reaction rates and avoiding CH_4_ thermal decomposition.^21^ A cycling experiment consists of 5 consecutive steps as elaborated next. (1) The vertical-tube reactor is initially purged of pure Ar (COREGAS grade 5.0) under a 250 mL min^−1^ flow at room temperature. The reactor is then heated from ambient room temperature to *T* = 1173 K at a ramp of 57.1 K min^−1^. (2) Subsequently, the sample is reduced under CH_4_ gas (COREGAS grade 4.5). The reduction step is performed using a mixture of CH_4_ in Ar (8 vol% CH_4_) at a total flow rate of 250 mL min^−1^. The period for the reduction step is varied within *t* = 1–30 min. (3) Then the tube is again purged of Ar (250 mL min^−1^) for 15 min. (4) The oxidation step is initiated by delivering a mixture of CO_2_ (COREGAS grade 4.5) in Ar (4 vol% CO_2_) at a total flow rate of 250 mL min^−1^. The period for the oxidation step is set for *t* = 7 min and 15 min. (5) The tube is purged with Ar (250 mL min^−1^) for 15 min before the next cycle. The selected gas flow rates are the same as those used in ref. 21.

We change the time periods of the reduction step (*t* = 1, 3, 5, 7, 10, 20 and 30 min) and of the oxidation step (*t* = 7 and 15 min) when the number of the redox cycling (Nc) is 1, as shown in Table 1. For example, in the case of the cycling experiment with the reduction time of *t* = 5 min, this experiment has no oxidation step in step (4) and no Ar purge in steps (3) and (5). The experiment with the oxidation time of *t* = 7 min has the oxidation time of *t* = 7 min after steps (1)–(3) and has no Ar purge in step (5). In the case of *N*_c_ = 5, the time periods of the reduction and oxidation steps are 30 min and 15 min, respectively. We repeatedly conduct cycling experiments for each experimental condition, especially, in the case of the experiments with shorter time periods of the reduction step, as shown in Table 1. The average TPA (total production amount) values of H_2_ and CO gases are obtained by averaging the values of the amount of gas production time-integrated over each step or each cycle. All of the gas volumes are reported at 293 K and 1 atm.

**Table 1:** Experimental conditions in the cycling experiment. *N*_c_ shows the number of the redox cycling. Figures in the parentheses are the numbers of cycling experiments under the same condition

*N*_c_		Reduction step	Oxidation step
1	Duration (min)	1 (5)/3 (4)/5 (7)/7 (4)/10 (6)/20 (5)/30 (4)	7 (4)/15 (5)
5	Duration (min)	30 (4)	15 (4)

### Cycling experiment for preheated ceria powders

We examine cycling experiments for preheated ceria powders using the same experimental set-up involved with the electric IR furnace shown in Fig. 1. The ceria powders are preheated for *t* = 50 h by using the electric furnace. We use three kinds of the preheated ceria powders with the different preheated dwell temperatures at *T* = 1173, 1273 and 1373 K. The SSA values after the preheating treatment are shown in Fig. 4. The number of the redox cycling is fixed at 5. The temperature in the cycling experiment is 1173 K. One redox cycling consists of 5 consecutive steps: (1) Ar gas purge (15 min, Ar), (2) reduction step (30 min, 8 vol% CH_4_ in Ar), (3) Ar gas purge (15 min, Ar), (4) oxidation step (15 min, 4 vol% CO_2_ in Ar) and (5) Ar gas purge (15 min, Ar) before the next cycle. The total flow rate for each step is 250 mL min^−1^.

### Sample characterization

The ceria powder samples are analyzed using the SEM (Ultraplus, Zeiss, operated at 3 kV without any coating) to obtain the information about the ceria morphology. The ceria powders are observed by the XRD (D2 PHASER, Bruker Ltd) to obtain the information about the crystal structural and elemental analysis. The Scherrer equation is applied for the several intense peaks to determine an average grain size. The Brunauer–Emmett–Teller (BET) specific surface area is measured by N_2_ adsorption–desorption isotherms at 77 K, using a surface and porosity analyzer (TriStar II, Micromeritics Ltd). The average SSA value is obtained by averaging the corresponding SSA values for the same experimental condition.

## Results and discussion

### Heating experiment without syngas production

**Fig. 2:**
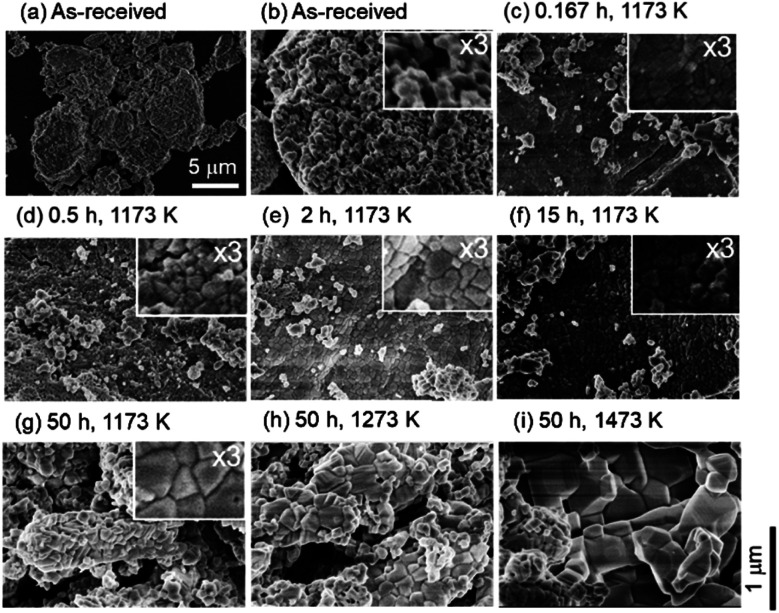
SEM images before (a) and (b) and after (c)–(i) the heating experiment without redox cycling. The magnifications of the SEM images (a) and (b)–(i) are ×10 000 and ×50 000, respectively. The spatial scale bars for (a) and (b)–(i) are indicated in (a) and at the lower right of this figure, respectively. The inseted images are ×3 magnified from the corresponding original SEM images.

Fig. 2 shows the SEM images of the ceria samples before Fig. 2(a) and (b) and after Fig. 2(c)–(i) the heating experiment without syngas production. Pure ceria powders before the experiment were agglomerates with a typical size of 5–10 μm (Fig. 2a). Furthermore, these agglomerates consisted of numerous small particles with a size of several hundreds of nanometers (see the inset in Fig. 2b). Particle size did not significantly change with increasing the heating time from *t* = 10/60 (0.167) h (in the inset of Fig. 2c) to *t* = 15 h (in the inset of Fig. 2f). On the other hand, larger agglomerates can be clearly seen at the heating time of *t* = 50 h (in the inset of Fig. 2g) and at the higher temperatures (in Fig. 2h and i).

**Fig. 3:**
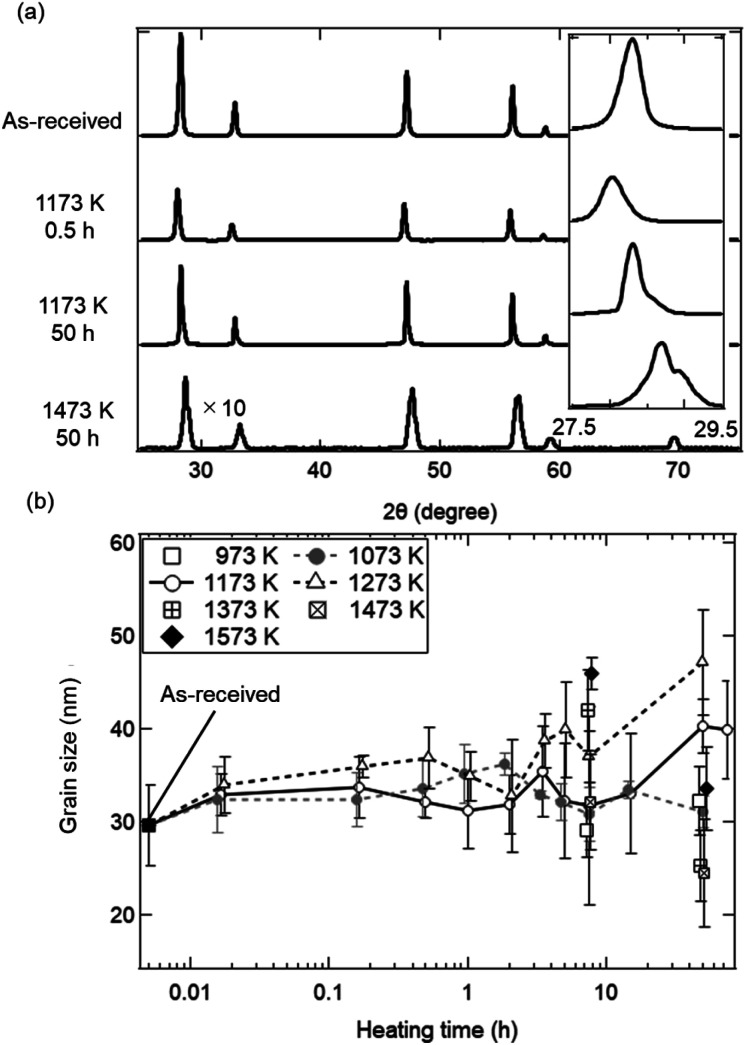
Crystal structural and elemental analysis using (a) X-ray spectra and (b) grain sizes as functions of temperature and heating time. (a) Four spectra before and after the heating experiment. The most intense peaks from 27.5–29.5 degrees are shown in the inseted figure. (b) In the cases of *T* = 1073, 1173 and 1273 K, grain sizes are obtained at a heating time of *t* = 0.0167 (1/60) h (1 min), 0.167 (10/60) h (10 min), 0.5 h (30 min), 1 h (60 min), 2 h, 5 h, 7.5 h, 15 h, 50 h and 75 h. In other temperatures, grain sizes at *t* = 7.5 h and 50 h are obtained. The initial grain size before the heating treatment is plotted at *t* = 0.005 h. Grain sizes at the same temperature are laterally displaced in order not to overlap each other.

To understand the evolution of structures during the heating experiment without syngas production, XRD analysis with respect to heating time and temperature was performed, as shown in Fig. 3a. All of X-ray peaks corresponded to those originating from pure ceria (see Fig. 3a). As shown in the inseted figures in Fig. 3a, the most intense peak at *T* = 1473 K and *t* = 50 h clearly shifted toward higher 2*θ* angle by 0.4 degrees from that of the as-received ceria, and had an additional new peak (peak shoulder). The peak shift and the peak shoulder would be attributed to the lattice contraction of ceria and to the ceria reduction, respectively.^34^ From Fig. 3b, the grain sizes were estimated to be ∼35 nm at *T* = 973 and 1073 K, and were almost constant. At *T* = 1173 and 1273 K, the grain sizes increased from the initial grain size (∼30 nm) to ∼40 nm and ∼47 nm, respectively, with increasing heating time from *t* = 0 h to *t* = 45 h. These observations were caused by grain growth at high temperatures.^22,24^ Note that the original grain size of ∼30 nm for the as-received samples was obtained by averaging the grain sizes for the 23 as-received samples.

**Fig. 4:**
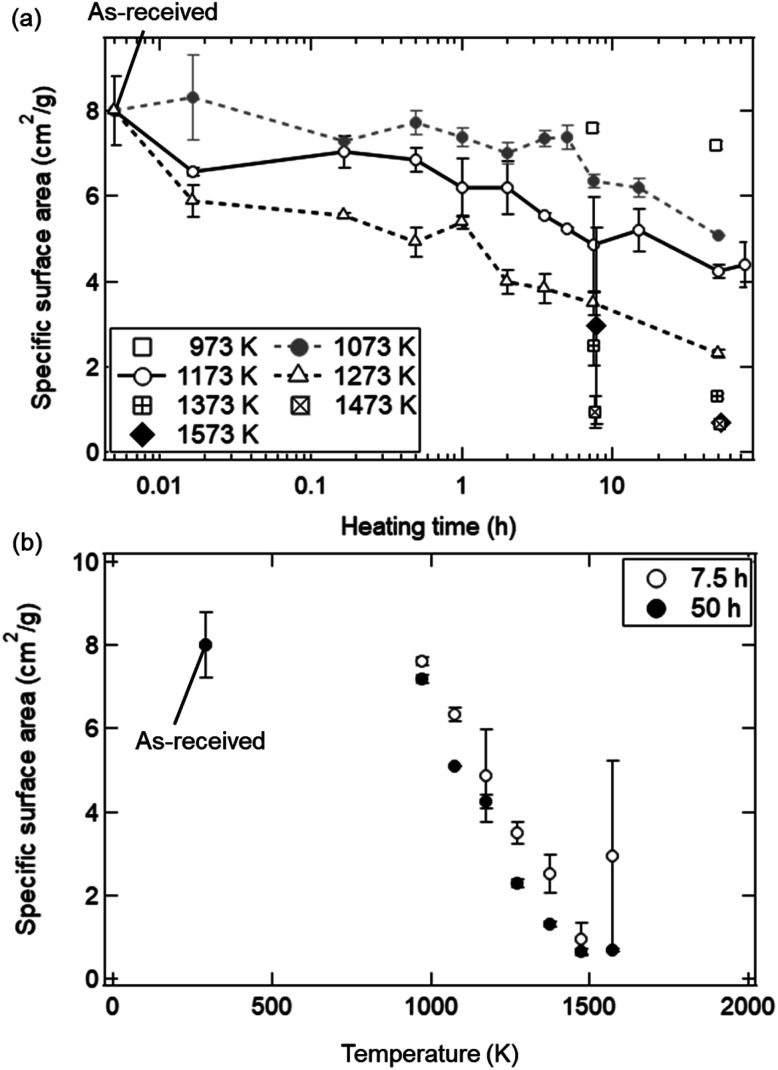
SSA results as functions of (a) heating time and (b) temperature. (a) In the cases of *T* = 1073, 1173 and 1273 K, the SSA values are shown at a heating time of *t* = 0.0167 (1/60) h (1 min), 0.167 (10/60) h (10 min), 0.5 h (30 min), 1 h (60 min), 2 h, 5 h, 7.5 h, 15 h, 50 h and 75 h. In other temperatures, the SSA values at *t* = 7.5 and 50 h are plotted. The initial SSA value before the heating treatment is plotted at *t* = 0.005 h. SSAs at *t* = 7.5 h are laterally displaced in order not to overlap each other. (b) SSA values at *t* = 7.5 and 50 h in a temperature range of *T* = 973–1573 K. The original SSA value for the as-received samples was plotted at *T* = 293 K.

The dependences of heating time and temperature on SSA are shown in Fig. 4. All of the SSA values decreased with increasing heating time and temperature. From Fig. 4a, the SSA value slightly decreased from ∼8.0 to ∼6.5 cm^2^ g^−1^ even at *t* = 1 min and *T* = 1173 K, and was almost constant during *t* = 1–30 min. The average SSA value during *t* = 1–30 min was 6.82 ± 0.24 cm^2^ g^−1^, and was used as an initial SSA value (SSA(0)) expressed in the eqn (6) and (7). The final SSA values at *t* = 50 h were 4.25 ± 0.15 cm^2^ g^−1^ (∼50% SSA of the as-received samples) at *T* = 1173 K, 2.30 ± 0.10 cm^2^ g^−1^ (∼25%) at *T* = 1273 K and 1.31 ± 0.08 cm^2^ g^−1^ (∼12.5%) at *T* = 1373 K, respectively. As seen in Fig. 4b, the SSA slightly decreases from ∼8.0 to ∼7.6 cm^2^ g^−1^ even at *T* = 973 K and *t* = 7.5 h. At higher temperatures, sample powders were observed to partially attach onto the crucibles due to sintering, leading to large variation of SSA at *T* = 1573 K.

As shown in Fig. 3b, the grain sizes did not markedly increase during a heating time of *t* = 1/60 to 50 h around *T* = 1173 K in the heating experiment without syngas production. These experimental results observed with XRD are available in the estimation of sintering extent in a late period with heating time from ∼2 to ∼100 h.^27–29,35^ The familiar grain growth equation in the late period is expressed by:

5 *G*^*n*^ − *G*_0_^*n*^ = *K*(*t* − *t*_0_)

where *G* is an average grain size at time *t*, *G*_0_ is an average initial grain size at time *t*_0_ = 0, *n* is a grain growth exponent and *K* is a characteristic material constant. It has been reported that grain growth is governed by a parabolic grain growth (*n* = ∼2–3) above ∼1573 K, and that lower *n* values at high temperatures lead to enhanced grain growth and larger grain size.^27,29^ On the other hand, experimentally grain growth exponent up to *n* = 10 were previously reported.^25,26,28^

**Fig. 5:**
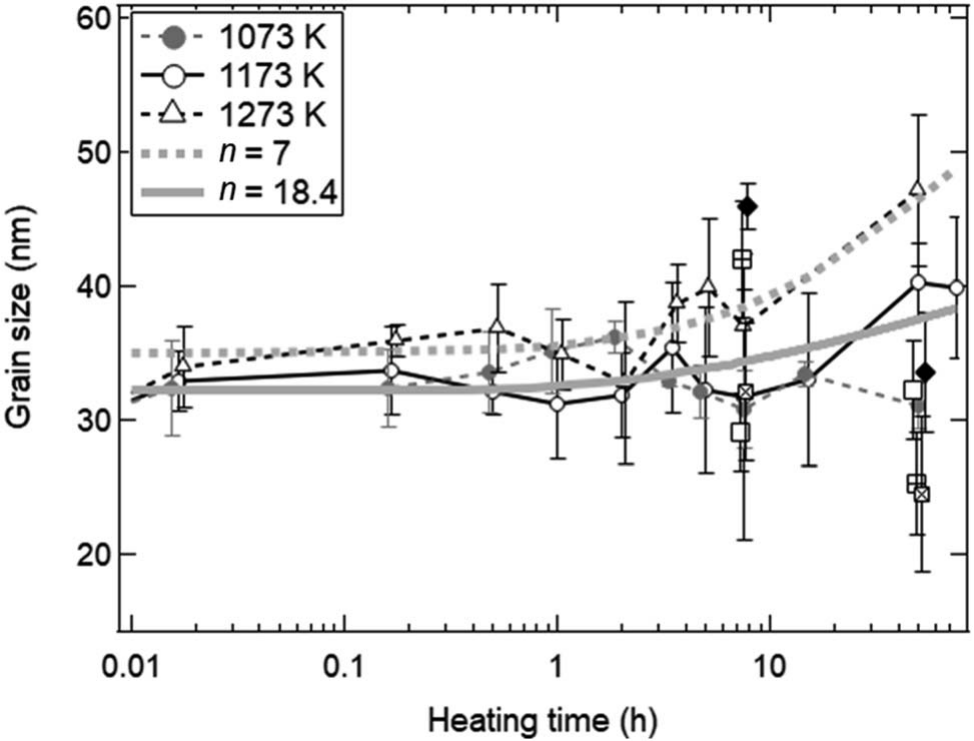
Estimation of a grain growth exponent n for grain growth without syngas production in the heating experiment without redox cycling. The experimental results shown in Fig. 3b are fitted by the kinetic equation on grain growth. The fitted solid and dotted curves are for *n* = 18.4 and 7, respectively.

Fig. 5 shows the fitted curves for *n* = 7 and 18.4 using the eqn (5). The fitted curves for *n* = 7 and 18.4 were in agreement with the temporal evolutions of grain sizes at *T* = 1273 and 1173 K, respectively. Note that the temporal evolution of grain growth at *T* = 1073 K could not be fitted using the eqn (5) because of no grain growth. A quite higher *n* value around 18 was observed at *T* = 1173 K, meaning the strong suppression of grain growth in the late period from ∼2 to ∼100 h. This can be observed in ceria samples or other materials at lower temperatures below *T* = 1373 K, and is called the self-limited grain growth.^28,35–37^ Grain growth ceases due to some factors such as impurities or pores when the self-limited grain growth is dominant at low temperatures. On the other hand, thermochemical redox cycling coupled to the reduction step at higher temperatures above *T* = ∼1500 K would result in a grain growth because of lower *n*.^26^ Thus, the sintering extent of ceria powders for the isothermal redox cycling at *T* = 1173 K coupled to MPO in this paper can be expected to be considerably suppressed from that of redox cycling using temperature swings with the reduction step higher than *T* = 1500 K.

### Isothermal redox cycling experiment with syngas production

**Fig. 6:**
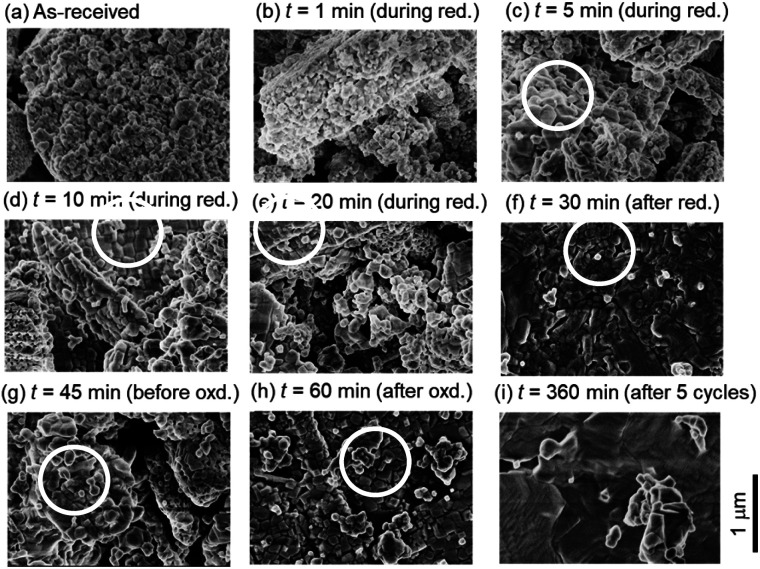
SEM images (a) before the isothermal redox cycling and (b)–(i) after the isothermal redox cycling at *T* = 1173 K. SEM images (b)–(f) during the reduction step, (g) and (h) during the oxidation step and (i) after the 5th cycle, respectively. The magnification of the SEM images is ×50 000. The spatial scale bar is indicated at the lower right of this figure.

Fig. 6 shows the SEM images of ceria powders after the isothermal redox cycling experiment. Fine structures with a size less than ∼100 nm were observed in Fig. 6a (before experiment) and Fig. 6b (*t* = 1 min). On the other hand, sintered structures with some angular shapes were recognized in the circles in Fig. 6c–h. Ceria powders started to sinter only in 5 min from the beginning of the reduction step. Furthermore, the ceria samples had a flat-like surface without fine structures at *t* = 360 min (see Fig. 6i).

**Fig. 7:**
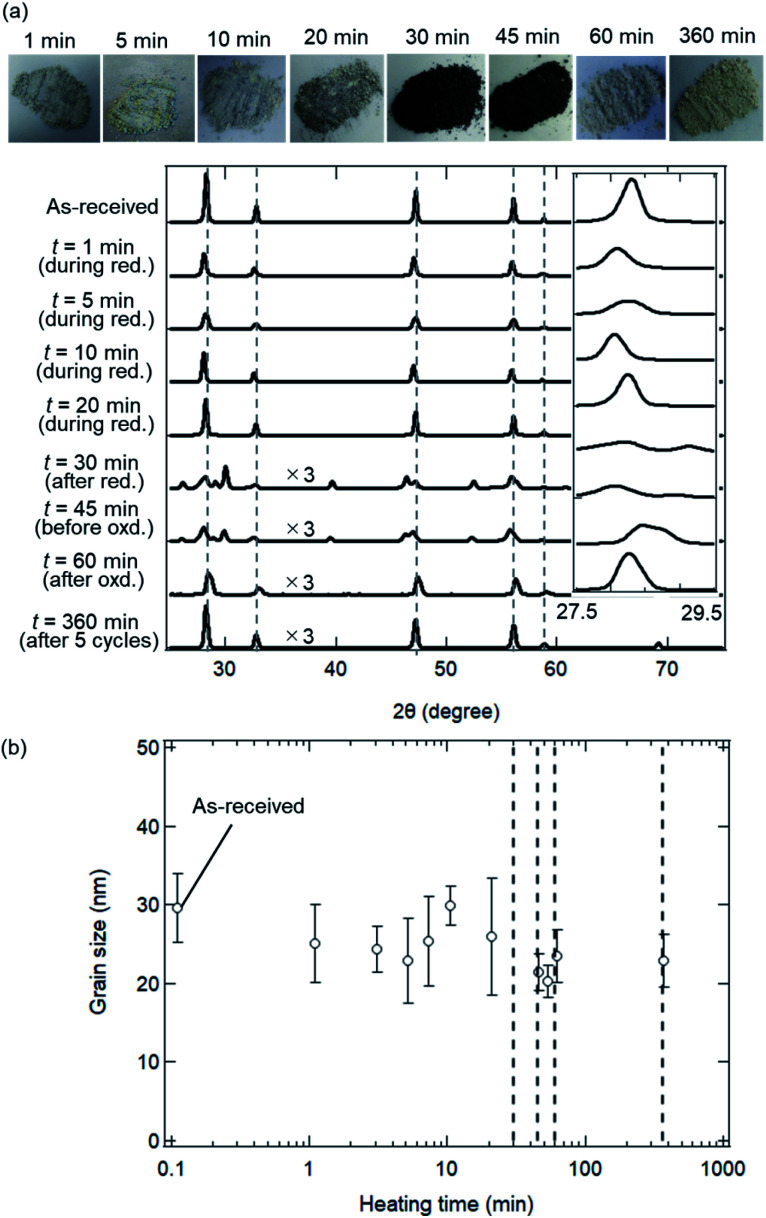
Crystal structural and elemental analysis using (a) X-ray spectra and (b) grain size as a function of heating time in the isothermal redox cycling experiment at *T* = 1173 K. (a) Nine spectra for the as-received samples, for the reduction step (*t* = 1, 5, 10, 20 and 30 min), for the oxidation step (*t* = 45 and 60 min) and after the 5th cycle (*t* = 360 min). Some spectra are displayed by ×3 magnified vertical axis compared to the original axis. Vertical dotted lines show typical X-ray peaks originating for ceria (CeO_2_). The most intense peaks from 27.5–29.5 degrees are shown in the inserted spectra. Photographs of the ceria powders after the redox cycling experiment are shown above the upper graph. (b) Dependence of heating time on grain size from *t* = 1 min to 360 min. The initial grain size before the redox cycling is plotted at *t* = 0.1 min. Vertical dotted lines at *t* = 30, 45, 60 and 360 min show the end of the reduction step, the beginning of the oxidation step, the end of the 1st cycle and the end of the 5th cycle, respectively.

Fig. 7a shows X-ray spectra during the reduction step (*t* = 1, 5, 10, 20 and 30 min), for the beginning and end of the oxidation step (*t* = 45 and 60 min) and for the end of 5th cycle (*t* = 360 min). All peaks in the X-ray spectra at *t* = 1–20 min in the reduction step corresponded to those of the as-received samples. It can be seen from the photographs of the examined powder samples that the colours gradually changed from white to grey during *t* = 1–20 min. X-ray spectra at *t* = 30 and 45 min were totally different from that of the as-received samples, and had new some peaks around 27–30 degrees, originating from the reduced ceria.^34^ The colours of the ceria samples became dark grey during *t* = 30–45 min. X-ray spectra at *t* = 60 and 360 min after the oxidation step in the 1st and 5th cycles returned back to the spectrum, which is similar to that of the as-received samples. On the other hand, the grain sizes were almost constant over *t* = 1–360 min (see Fig. 7b). The colours also returned back to the original white colour at *t* = 60 min and 360 min (just after the oxidation step). Returning to the original colour after the oxidation step means that carbons did not deposit on the surface of the ceria samples in the present experiment.

**Fig. 8:**
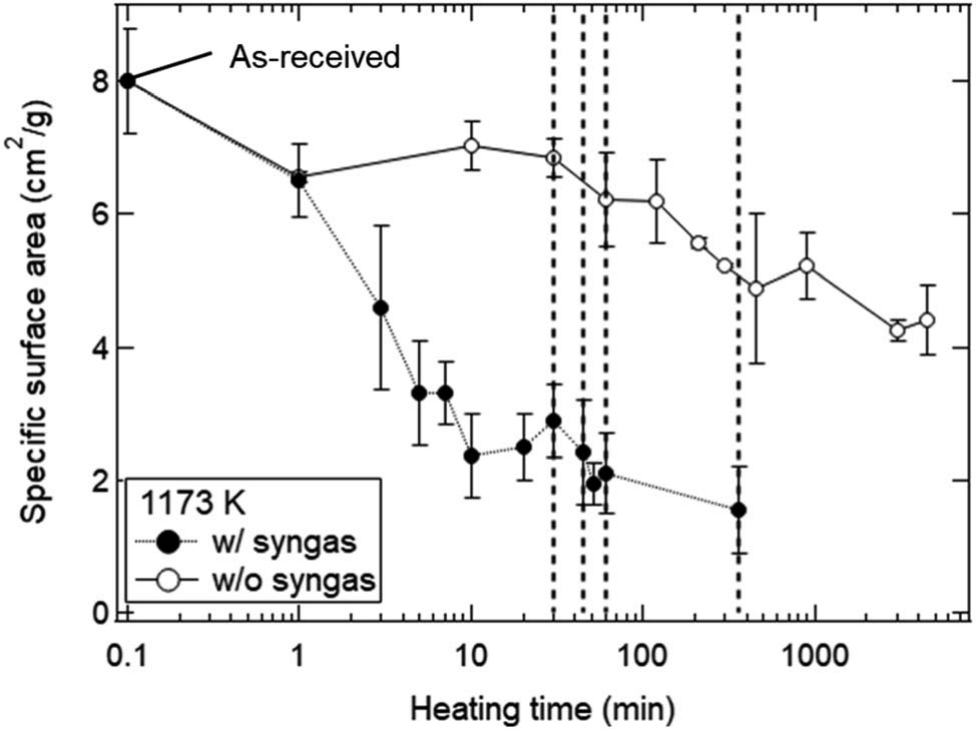
Dependence of heating time on SSA. Closed and open circles indicate the SSA values for the isothermal redox cycling with syngas production and for the heating experiment without syngas production, respectively, at *T* = 1173 K. The initial average SSA value for the as-received samples before the redox cycling is plotted at *t* = 0.1 min. Vertical dotted lines at *t* = 30, 45, 60 and 360 min show the end of the reduction step, the beginning of the oxidation step, the end of the 1st cycle, and the end of the 5th cycle, respectively.

Fig. 8 shows the dependence of heating time on SSA in the isothermal redox cycling experiment at *T* = 1173 K. The SSA values in the isothermal redox cycling experiment rapidly decreased from ∼6.5 to ∼2.5 cm^2^ g^−1^ within a range of *t* = 1–10 min compared to the SSA values in the heating experiment without syngas production even when the self-limited grain growth was dominant at low temperatures (around *T* = 1173 K) as shown in Fig. 5. The SSA value was almost constant at ∼2 cm^2^ g^−1^ after *t* = 10 min. Note that the both SSA values with and without syngas production at *t* = 1 min were mostly similar to each other.

**Fig. 9:**
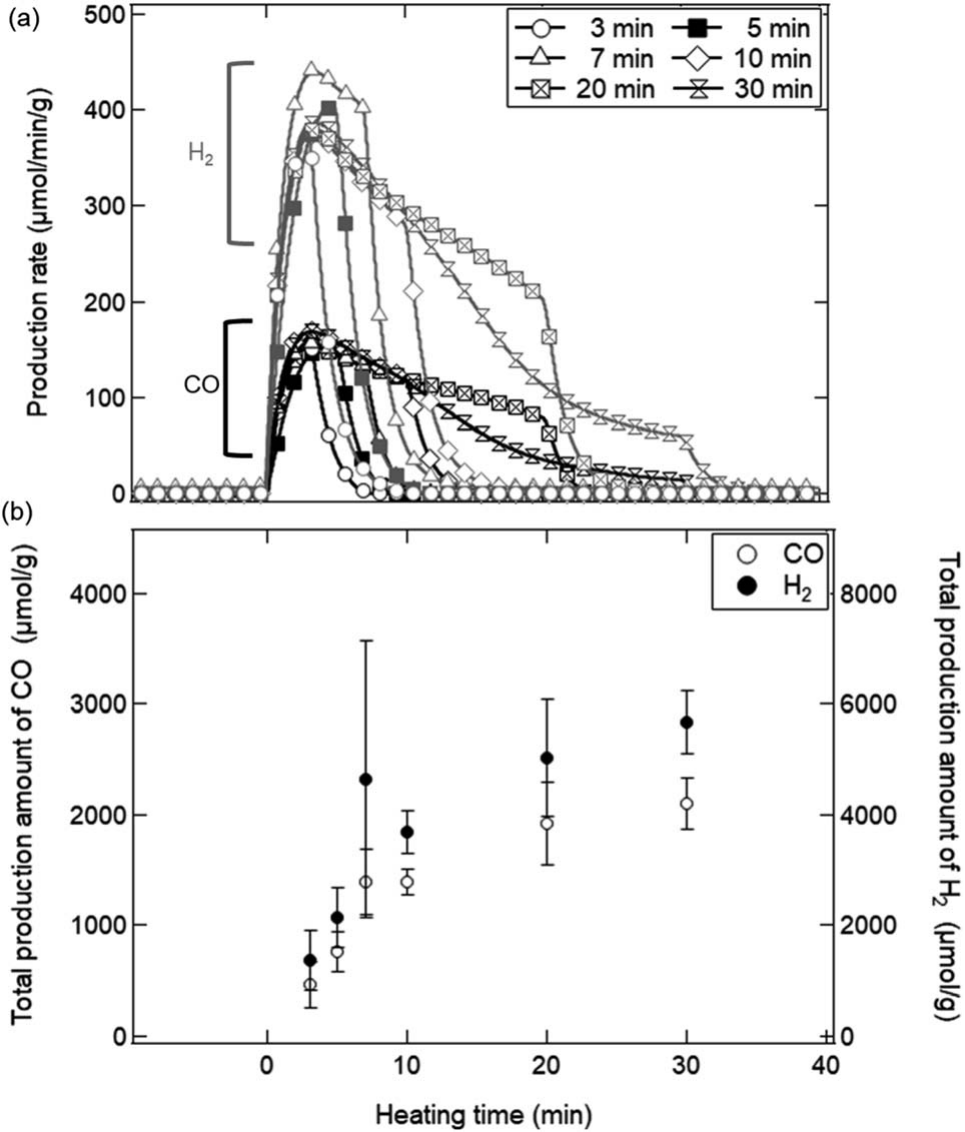
(a) Production rates of H_2_ and CO gases in the reduction step during *t* = 0–30 min. (b) Total production amount of H_2_ and CO in the reduction step.

Fig. 9a shows a typical temporal behaviour of syngas production rate (SPR) in the reduction step (*t* = 0–30 min). H_2_ and CO gases generated around a ratio of 2 : 1 in the reduction step. H_2_ gases shown by grey symbols rapidly started to increase from *t* = 0 min, and reached the maximum SPR value around 400 μmol min^−1^ g^−1^. H_2_ gases continued to generate during heating, and ceased after heating. CO gases shown by black symbols generated as well as those for H_2_ gases. Fig. 9b shows the total production amount (TPA) of H_2_ and CO gases in the reduction step. For example, the TPA value at *t* = 10 min was calculated by time-integrating the SPR values over *t* = 0–10 min. From Fig. 9b, we can see that the TPA of CO and H_2_ gases significantly increased during the initial period of the reduction step until *t* = 10 min. This initial period would mostly correspond to the time period when SSA rapidly decreased as shown in Fig. 8. A relative slight increase of TPA was observed after *t* = 10 min.

**Fig. 10:**
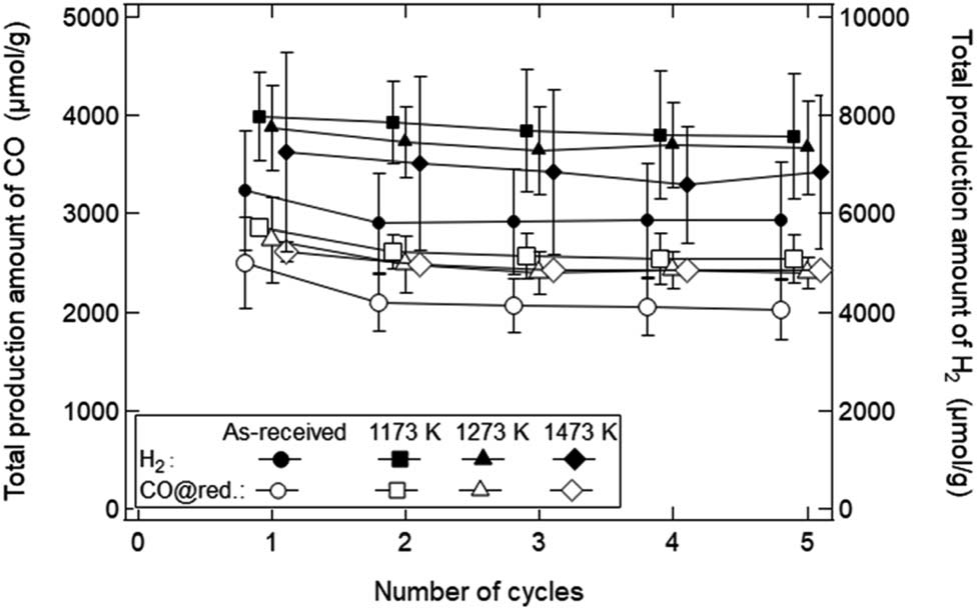
Total production amount of CO and H_2_ gases only in the reduction step for the preheated ceria samples. The TPA values of H_2_ and CO gases are shown by closed and open symbols, respectively. Circular symbol represents the data for the as-received sample. Square, triangle, and diamond symbols show the data of the samples preheated at *T* = 1173, 1273, and 1473 K, respectively. The data at the same number of cycles are slightly shifted and plotted not to overlap with each other.

Fig. 10 shows the dependence of the number of cycles (*N*_c_) on the TPA of syngas in the reduction step for the ceria samples preheated at *T* = 1173, 1273 and 1473 K. The initial SSA values before the redox cycling experiment were 8.02 ± 0.80 cm^2^ g^−1^ for the as-received samples, 4.25 ± 0.15 cm^2^ g^−1^ (∼50% SSA of the as-received samples) at *T* = 1173 K, 2.30 ± 0.10 cm^2^ g^−1^ (∼25%) at *T* = 1273 K and 1.31 ± 0.08 cm^2^ g^−1^ (∼12.5%) at *T* = 1373 K, respectively, as shown in Fig. 4a. Note that CO production in the oxidation step was not taken into consideration when the TPA value of CO gas was calculated. The TPA values of CO and H_2_ gases for the as-received samples at *N*_c_ = 1 were slightly higher than those at *N*_c_ = 2–5, and were mostly constant after *N*_c_ = 2. Surprisingly, the TPA values of CO and H_2_ gases for the preheated ceria samples with lower SSA were higher than those for the as-received samples with higher SSA.

The sintering mechanism in the initial period can be analyzed using the information on the reduction of SSA.^38^ Here, the initial period corresponds to the time period when SSA decreases from the initial SSA to half of the initial SSA. In the present isothermal redox cycling experiment, the initial period is corresponding to the time period *t* = 1–10 min (see Fig. 8). The generalized kinetic equations at this stage can be expressed by:^38^

6 (ΔSSA(*t*)/SSA(0))^*γ*^ = *kt*

7 ΔSSA(*t*) = SSA(0) − SSA(*t*)

where SSA(*t*) is a specific surface area at time *t*, SSA(0) is an initial specific surface area at *t* = 0, *k* is a coefficient and *γ* is the exponent of SSA reduction. The *γ* value is related to sintering mechanism due to diffusive processes, and can be estimated to be ∼1.1 for viscous flow, ∼1.5 for evaporation condensation, ∼2.7 for volume diffusion, ∼3.3 for grain-boundary diffusion and ∼3.5 for surface diffusion, respectively.^38^ This model involves the following assumptions. (1) The particles are assumed to be monodisperse spheres. Large deviations in either particle shape or size distribution could produce unexpected effects that cannot be predicted by this model. (2) This model can be only accepted within the range up to ΔSSA(*t*)/SSA(0) ∼50% in the initial period of sintering. It should be emphasized that XRD and SEM observations are not available to the analysis of sintering mechanism in the initial period because their results did not significantly change in the initial period *t* = 1–10 min (see Fig. 6 and 7).

**Fig. 11:**
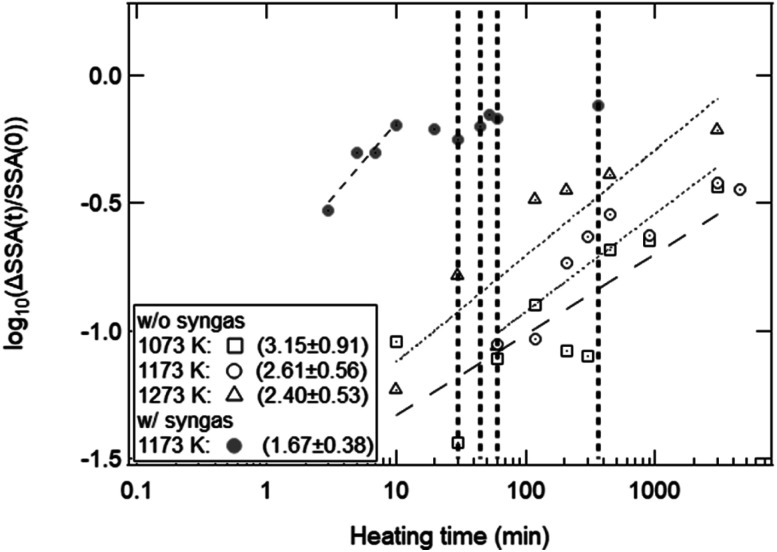
Estimation of the exponent *γ* for the heating experiment without syngas production at *T* = 1073, 1173, 1273 K and for the isothermal redox cycling experiment with syngas production at *T* = 1173 K. Each dotted line shows the fitted line for each temperature. The *γ* values for four experiments are shown at the lower left of this figure. 1st, 2nd, 3rd and 4th vertical dotted lines represent the end of the reduction step, the beginning of the oxidation step, the end of the 1st cycle and the end of the 5th cycle in the isothermal redox cycling experiment.

Fig. 11 shows the effect of heating time on the SSA reduction in the heating experiment without syngas production and in the isothermal redox cycling experiment with syngas production. The *γ* values in the heating experiment without syngas production were 3.15 ± 0.91 at *T* = 1073 K, 2.61 ± 0.56 at *T* = 1173 K and 2.40 ± 0.53 at *T* = 1273 K, respectively. The correlation coefficients of the fitted lines at *T* = 1073, 1173 and 1273 K were 0.794, 0.901 and 0.896, respectively. Note that the SSA reduction data were fitted in the initial period of ΔSSA(*t*)/SSA(0) < 50%. Here, as noticed previously, SSA(0) at *T* = 1173 K was defined as the SSA value at *t* = 1 min. For example, the curve fitting at *T* = 1173 K for the isothermal redox cycling experiment was carried out using the SSA data at *t* = 1–10 min. The SSA(0) values at *t* = 1 min for the heating experiment without redox cycling and for the isothermal redox cycling experiment were mostly the same, as shown in Fig. 8. This SSA reduction from the initial SSA to SSA(0) at *t* = 1 min might be caused due to sintering during temperature rise from room temperature to *T* = 1173 K. Thus, the influence of the initial sintering during the temperature rise might be eliminated in the estimation of the *γ* value.

As described before, the *γ* values of the heating experiment without syngas production and of the isothermal redox cycling with syngas production were ∼2.40–3.15 and ∼1.67, respectively. These *γ* values had a large estimation error because the observed.

SSA values had large measurement error. However, a clear difference of the *γ* value between the heating experiment without syngas production and the isothermal redox cycling was observed. Therefore, we believe that the corresponding sintering mechanisms in the both experiments without and with syngas production would be different. It has been reported that single crystal ceria microspheres sinter mainly due to grain-boundary diffusion with *γ* = ∼3.3.^38^ The observed *γ* values for the heating experiment were ∼2.40–3.15, and were slightly lower than the *γ* value for single crystal ceria microspheres. Bouala *et al.* showed that polycrystalline microspheres easily sintered compared to single crystal microspheres due to oriented attachment (OA).^32^ At the beginning of heating, several crystallites with nanoscale size are present in the region of contact between the two grains. The formation of the neck is then probably driven by the reorganization of crystalline planes in the same orientation, which promotes the formation of a continuous lattice between the grains. This crystalline plane reorganization then leads to the formation of the neck between the grains constituted by many crystallites. Such a mechanical rearrangement for ceria has a low activation energy compared to single crystal microspheres. Thus, we believe that the *γ* values obtained in the heating experiment without syngas production were lower than the *γ* value shown in ref. 38, because our ceria powder samples with complex and fine morphologies would sinter due to a combination of OA and grain-boundary diffusion.

On the other hand, the *γ* value at *T* = 1173 K in the isothermal redox cycling experiment with syngas production was 1.67 ± 0.38 as shown in Fig. 11. This *γ* value is close to that of viscous flow (∼1.1) or that of evaporation condensation (∼1.5). The ceria is reduced *via* MPO as follows:^21^

8 CeO_2_ + *δ*CH_4_ → CeO_2−*δ*_ + *δ*CO + 2*δ*H_2_

The CO released during the reduction step results from the formation of O-vacancies within the ceria material. The decrease in the surface area and crystallite growth occurs *via* atomic transport at high temperatures, especially, the O^2−^ ion transport through the ceria material.^1,8^ Thus, the ceria is reduced through the combined process of atomic diffusion within the ceria (at solid phase) and the formation of O-vacancies for CO release (at gas phase). Therefore, we presume that the decrease in SSA at the initial period of the reduction step would be mainly caused due to evaporation condensation. This assumption is consistent with the results on syngas production rate (SPR) as shown in Fig. 9. Both the SSA and SPR values rapidly changed in this initial period (*t* = 0–10 min). It can be expected that an enhanced syngas production in this initial period would be mainly caused by the formation of the O-vacancies in the ceria samples, resulting in the observed rapid decrease in SSA due to evaporation condensation. This detailed investigation on sintering mechanism is beyond the scope of this paper. Note that the observed rapid decrease of SSA might be affected by phase change from CeO_2_ to CeO_2−*δ*_. As shown in Fig. 7a, new X-ray peaks in X-ray spectra were able to observe only after the initial period (after *t* = 10 min). This implies that the phase change might have a negligible effect upon the observed rapid decrease of SSA.

As explained before, we found that the SSA values rapidly and markedly decrease in the initial period of the reduction step (*t* = 0–10 min). As shown in Fig. 10, the total amount of syngas production for the as-received samples with the highest initial SSA values were lower than those for the preheated samples with lower initial SSA values. This means that an initial SSA has no strong influence on the total amount of syngas production in a long-term period because the SSA value rapidly and markedly decreases in the initial period of the reduction step in the 1st cycle. Furthermore, it should be emphasized that this rapid decrease in SSA was observed in the isothermal redox cycling even at lower temperatures. This lower temperature leads to the self-limited grain growth in the late period of sintering observed in the heating experiment without syngas production, as shown in Fig. 5. Namely, a rapid decrease in SSA with syngas production would be mainly caused due to evaporation condensation even when the self-limited grain growth in the late period of sintering is dominant at low temperatures.

**Fig. 12:**
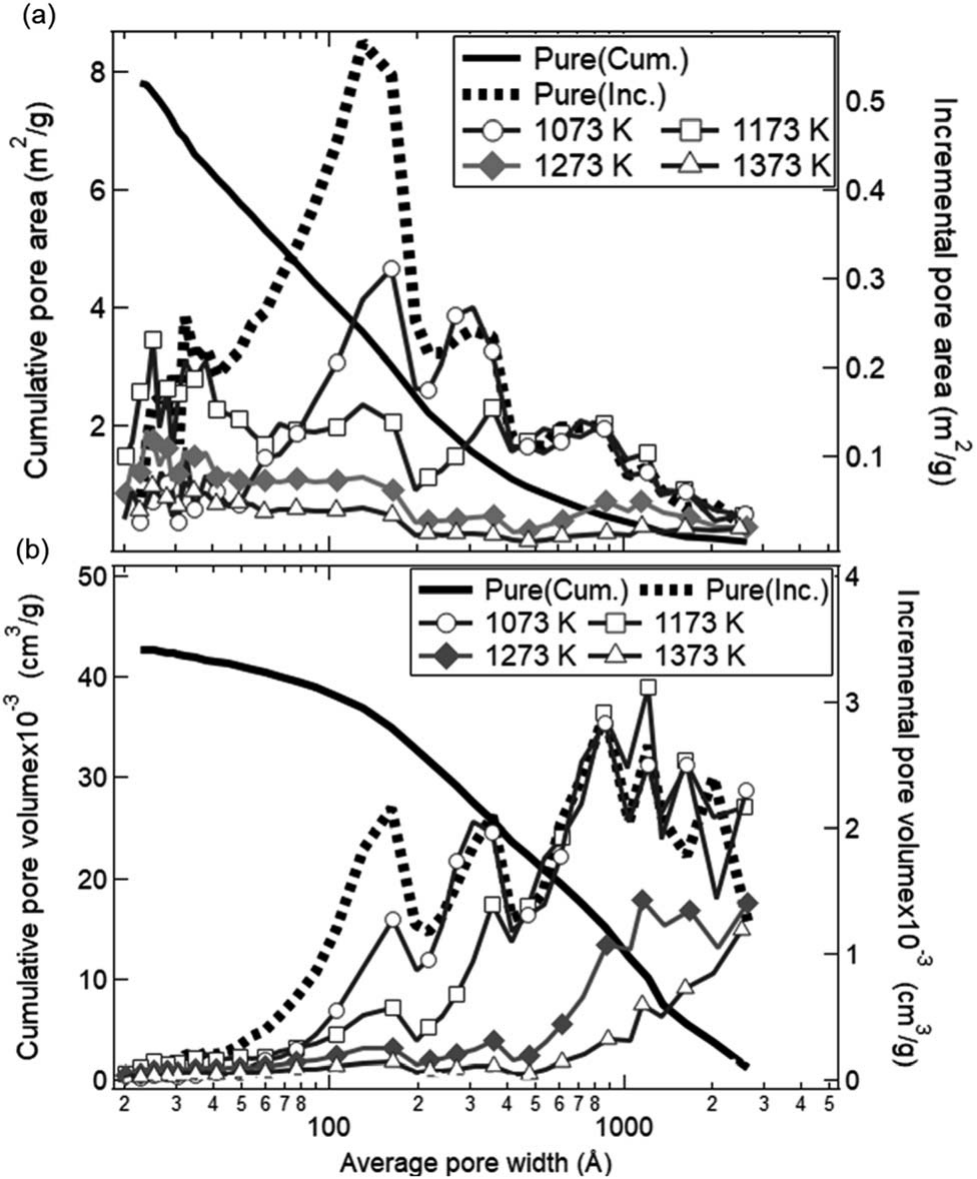
Dependences of average pore width on (a) BJH pore area and (b) BJH pore volume observed with the BET equipment. Dotted and solid curves show the dependences of the incremental and cumulative data for as-received ceria powder samples as a function of the average pore width, respectively. The incremental pore areas and pore volumes for the ceria preheated at *T* = 1073, 1173, 1273 and 1373 K are shown by circular, square, diamond and triangle symbols, respectively. The preheating time is 50 h.

Fig. 12 shows the dependences of an average pore width on BJH pore areas and BJH pore volumes observed with the BET equipment. As shown by the solid black line, the cumulative pore area integrated over the average width was identical with the initial *S*_BET_ value (8.02 ± 0.80 cm^2^ g^−1^) for the as-received ceria powder samples. The as-received ceria powder samples had a higher pore area and pore volume for the average pore width of ∼10–20 nm and 10–300 nm, respectively. On the other hand, in the cases of all the preheated ceria samples, the pore areas decreased over the whole range of the average pore width. The pore volumes also decreased below 100 nm with an increase of preheating temperature. As shown in Fig. 10, the total production amount of syngas for the sample preheated at *T* = 1173 K was higher than that of the as-received samples. This implies that pores with a width less than 100 nm might not be available for an efficient and long-term syngas production. Thus, we believe that relative larger structures of the order of micron would be essential for long-term and stable performances in syngas production under the conditions examined in this study.

## Conclusions

In this study, we have experimentally investigated the effect of specific surface area on syngas production of pure ceria powders under two experiments such as the heating experiment without redox cycling (without syngas production) and the isothermal CDS–MPO redox cycling experiment (with syngas production). The temperatures of the reduction and oxidation steps were fixed at *T* = 1173 K in the isothermal CDS–MPO redox cycling. We have studied the sintering mechanism and the sintering extent of the ceria powders using the information on the reduction of SSA and the growth of grain size, respectively. In the case of sintering without syngas production, the SSA of ceria powders decreased relatively slowly due to a combination of oriented attachment and grain-boundary diffusion. In the case of sintering with syngas production at lower temperatures (around *T* = 1173 K), the SSA of ceria powders rapidly decreased only in the initial 10 min of reduction in the 1st cycle due to evaporation condensation. Thus, it is expected that a significant decrease of SSA during the initial stage of thermochemical redox cycling using ceria powders is unavoidable even if lower temperatures (around *T* = 1173 K) are used by introducing MPO to the reduction step.

## Conflicts of interest

The authors declare no conflict of interest.

## Supplementary Material
